# Calcium Signaling Is Required for Erythroid Enucleation

**DOI:** 10.1371/journal.pone.0146201

**Published:** 2016-01-05

**Authors:** Christina B. Wölwer, Luke B. Pase, Sarah M. Russell, Patrick O. Humbert

**Affiliations:** 1 Cell Cycle and Cancer Genetics, Peter MacCallum Cancer Centre, Melbourne, Victoria, Australia; 2 Sir Peter MacCallum Department of Oncology, University of Melbourne, Melbourne, Victoria, Australia; 3 Immune Signaling Laboratory, Peter MacCallum Cancer Centre, Melbourne, Victoria, Australia; 4 Centre for Micro-Photonics, Faculty of Engineering and Industrial Sciences, Swinburne University of Technology, Melbourne, Victoria, Australia; 5 Department of Pathology, University of Melbourne, Melbourne, Victoria, Australia; 6 Department of Biochemistry and Molecular Biology, University of Melbourne, Melbourne, Victoria, Australia; Institut de Génétique et Développement de Rennes, FRANCE

## Abstract

Although erythroid enucleation, the property of erythroblasts to expel their nucleus, has been known for 7ore than a century, surprisingly little is known regarding the molecular mechanisms governing this unique developmental process. Here we show that similar to cytokinesis, nuclear extrusion requires intracellular calcium signaling and signal transduction through the calmodulin (CaM) pathway. However, in contrast to cytokinesis we found that orthochromatic erythroblasts require uptake of extracellular calcium to enucleate. Together these functional studies highlight a critical role for calcium signaling in the regulation of erythroid enucleation.

## Introduction

Erythroid enucleation is the process by which the future red blood cell disposes of its nucleus prior to entering the blood stream. This key event in red blood cell development shares many similarities with cytokinesis [1]. Of note, calcium is a universal signaling molecule, involved in multiple cellular processes, including cytokinesis [2]. Studies in Xenopus or zebrafish embryos have documented transient Ca^2+^ waves preceding cytokinesis [3, 4], and Ca^2+^-chelators, such as BAPTA, inhibited cytokinesis [3, 5]. To our knowledge a Ca^2+^ wave has not been reported for erythrocyte enucleation, although Yoshida et al observed increased calcium levels in nuclei of enucleating erythroblasts [6]. Here we have used live cell imaging together with functional studies and demonstrate for the first time that a Ca^2+^ flux occurs prior to enucleation, and show an absolute requirement for external calcium and signaling through the Calmodulin (CaM) pathway for nuclear extrusion during the process of erythroid enucleation.

## Materials and Methods

### Materials

Phenylhydrazine hydrochloride was purchased from Aldrich Chemistry. CD44- PE-Cy7 anti-mouse antibodies and the BrdU Flow Kit were purchased from BD Pharmingen. Ter119-Alexa Fluor 647 anti-mouse antibodies were purchased from Biolegend. Hoechst 33342 was purchased from Invitrogen. Propidium iodide (PI) was purchased from Merck. Dimethyl sulfoxide (DMSO) was purchased from Calbiochem. Rapid Diff stain was purchased from Australian Biostain. β-actin antibodies, Cytochalasin D, Bapta-AM, KB-R7943, Thapsigargin, CGS-9343B, W-7, KN-62 and KN-93 were purchased from Sigma Aldrich. Calmodulin (FL-149) and NCKX1 (E-6) antibodies were purchased from Santa Cruz. Fluo-3 was purchased from Molecular Probes/Life Technologies. Microgrids [7] purchased from Microsurfaces Pty Ltd.

### Animal experiments and orthochromatic erythroblast isolation

All animal procedures were approved by the Peter MacCallum Cancer Centre Animal experimentation Ethics Committee. To induce stress erythropoiesis, C57Bl/6 mice at 6–12 weeks of age were administered intraperitoneal injections of phenylhydrazine hydrochloride (60μg/g) on day 0 and day 1 of the experiments. On day 4 of the experiments, cells were harvested from mouse spleens and stained for Hoechst, CD44 and Ter119. All Hoechst negative (enucleated) cells were excluded from the sort. Orthochromatic erythroblasts were isolated based on their Ter119 and CD44 expression by FACS Aria ΙΙ special order system (BD) using the FACS Diva software (BD). PI was used to exclude dead cells from the sort. Orthochromatic erythroblasts (30,000 cells/well) were incubated in 96-well plates in the presence of the individual compounds in a final volume of 200μl/well for 5h at 37°C. Enucleation was quantified by FACS LSR ΙΙ (BD) using the FACS Diva software (BD). Net percentage of enucleation was derived by dividing the number of enucleated cells (Ter119^+^/Hoechst ^-^) by the sum of enucleated cells and erythroblasts (Ter119^+^/Hoechst^+^), and by multiplying the quotient by 100. PI was used to exclude dead cells from the analysis.

### Cytospins

30,000–60,000 orthochromatic erythroblasts were spun onto slides at 320rpm for 4min. Slides were air-dried before fixing with MeOH, stained with Rapid Diff and quantitated manually under the microscope (Olympus BX-51, 100x/1.40 NA oil objective).

### Immunofluorescence

3 x 10^4^ cells were allowed to settle on poly-L-lysine coated slides (Menzel-Glaeser, Thermo Scientific) for 30-60min at 37°C. Cells were fixed in 4% paraformaldehyde (Electron Microscopy Sciences) for 5min and subsequently permeabilized (0.1% Triton and 0.2% BSA in PBS) for 5min. Cells were then blocked in 1% BSA in PBS for 1h at RT prior to staining with the primary antibody at 4°C o/n. Cells were washed 3 times in 0.1% BSA in PBS prior to staining with the secondary antibody for 1h at RT. Cells were washed 3 times in 0.1% BSA in PBS prior to mounting in ProLong® Gold antifade reagent with DAPI (Invitrogen). Slides were sealed with nailpolish and stored at 4°C.

### Live cell imaging

To visualize calcium signaling during enucleation, FACS sorted orthochromatic erythroblasts were incubated in the presence of Fluo-3 (1.5mM) for 30min. Cells were than washed, resuspended in media and allowed to settle on microgrids in a microscopy chamber (ibidi) at 37°C and 5% CO_2_. Images were taken every minute for 2h using a Confocal Leica Sp5 microscope. Fluorescence intensity was analyzed using Image J.

## Results and Discussion

### Erythroid enucleation requires intracellular calcium signaling

**Fig 1:**
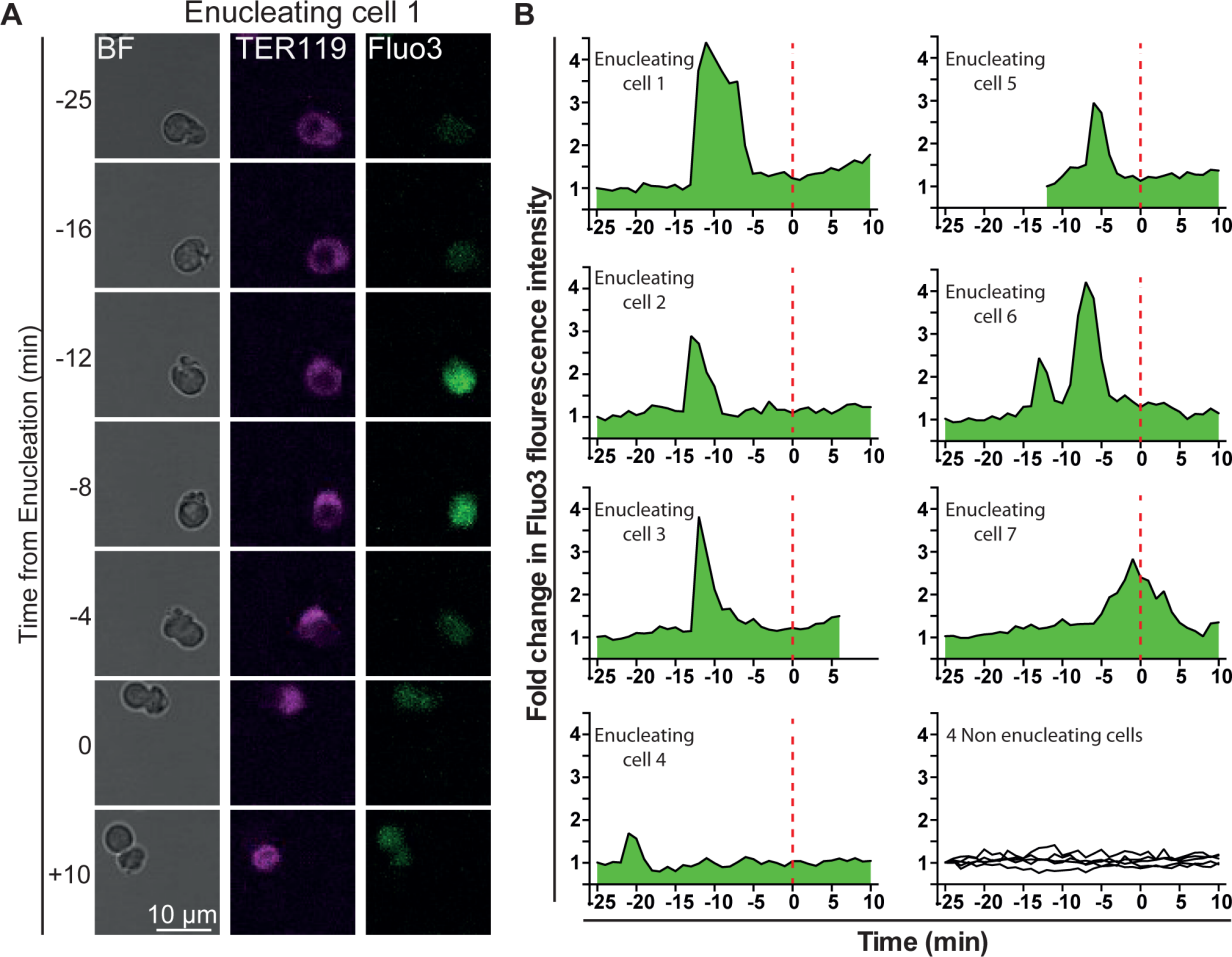
Orthochromatic erythroblasts display transient calcium bursts prior to enucleation. Orthochromatic erythroblasts were isolated by FACS and incubated in the presence of Fluo-3 for 30min. Orthochromatic erythroblasts were then washed and allowed to settle on a microgrid for live cell imaging using the Leica Confocal Sp5 microscope. Images were taken every minute for 2h. **(A)** Images of the same orthochromatic erythroblast at various time points prior (-), during (0) and after (+) nuclear extrusion. For the cell demonstrated here, Fluo-3 fluorescence is most intense between 12 and 8 min prior to nuclear extrusion. **(B)** Analysis of Fluo-3 fluorescence intensity in different enucleating cells compared to non-enucleating ones.

**Fig 2:**
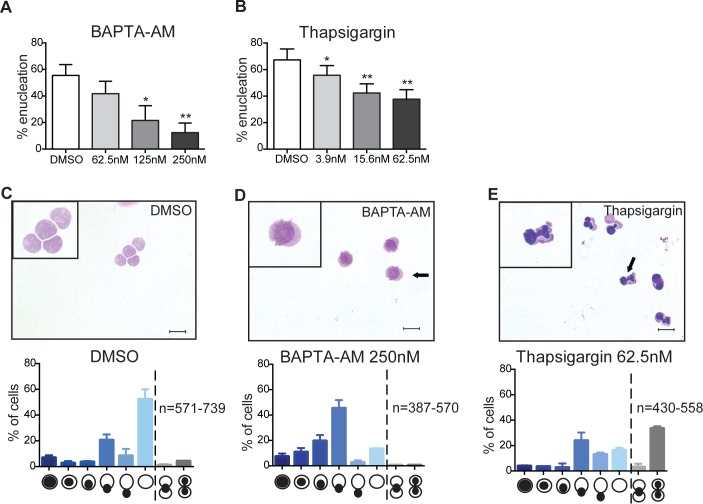
Erythroid enucleation requires intracellular calcium signaling. **(A-B)** Orthochromatic erythroblasts were FACS sorted and incubated in the presence of the compounds for 5h. Graphs showing percentages of enucleation in the presence of the indicated inhibitors at the indicated concentrations. Data are means (+/- SD) of 3 independent experiments analyzed using FACS LSR II (*P< 0.05, **P< 0.01, ***P< 0.001, ****P< 0.0001 (paired student’s t-test)). **(C-E)** Orthochromatic erythroblasts were incubated in media containing DMSO (vehicle control) or the indicated compounds at the indicated concentration for 5h and subsequently cytospun. For quantitative analysis cells were manually examined (Olympus BX-51 microscope; 100x/1.40 NA oil objective) using the Spot Advanced software (version 4.7)) and assigned a morphological class as per illustration. Data are means (+/- SD) of 3 independent experiments. Scalebar = 10μm.

Live cell imaging using the calcium sensor Fluo-3 indicates that a sharp calcium burst occurs throughout the orthochromatic erythroblasts 10 ± 6.3 minutes prior to nuclear extrusion (Fig 1), suggesting that calcium signaling may be involved in enucleation. Of note, enucleating orthochromatic erythroblasts were very sensitive to Fluo-3 concentrations used in the assay with high levels of Fluo-3 significantly impairing enucleation in these experiments likely due to caging effects, suggesting that tight control of intracellular calcium levels may be critical for enucleation. Based on these observations we explored the functional requirement of calcium in the enucleation event. Signaling pathways involving calcium are often of fast and short-lived dynamic nature. Similarly, erythroid enucleation is a 10min process [6] that is difficult to investigate by genetic methods, such as RNAi studies, due to the lag time between knockdown and the enucleation event, which allows for indirect effects of RNAi. Furthermore, experimental approaches combining *in vitro* erythropoiesis and inhibitors have also generated variable results due to the difficulty of synchronizing erythroblasts and the inability to exclude indirect effects of inhibitors on the proliferation of earlier erythroblast stages. We therefore utilized an approach that allowed us to directly question the pathways involved in the actual process of erythroid enucleation: orthochromatic erythroblasts were enriched *ex vivo* by FACS and subsequently exposed to known inhibitors for 5h. Enucleation was quantified using FACS analysis [8]. Dead cells were excluded from the analysis. We only used inhibitors and concentrations that are not toxic to the cells. Therefore, viability is normally at least 80%. Exposing orthochromatic erythroblasts to BAPTA-AM, a cell-permeable chelator of intracellular calcium, resulted in dose-dependent inhibition (up to 78% decrease) of enucleation (Fig 2A). The presence of Thapsigargin, a compound that traps and increases levels of calcium in the cytosol, by inhibiting ATP-dependent calcium uptake into intracellular stores, such as the endoplasmic reticulum (ER) [9], also resulted in a significant decrease (up to 44%) in enucleation efficiencies (Fig 2B). To morphologically characterize the effects of the inhibitors on enucleating erythroblasts, isolated orthochromatic erythroblasts were cytospun, imaged and quantitatively analyzed for distinct morphologies. An example of such an analysis in the presence of DMSO, the vehicle control, is illustrated in Fig 2C. Morphological analysis of cytospun erythroblasts revealed that in the presence of BAPTA-AM enucleating cells were arrested during nuclear extrusion (Fig 2D). Interestingly, up to 35% of cytospun cells allowed to enucleate in the presence of Thapsigargin showed a segmentation of their nucleus with discrete compartments connected by DNA bridges (Fig 2E), a phenotype that was not, or only rarely observed in the presence of the vehicle control or other inhibitors interfering with calcium signaling. Together, these results suggested that erythroid enucleation is sensitive to changes in intracellular calcium concentrations.

### Erythroid enucleation requires uptake of extracellular calcium

**Fig 3:**
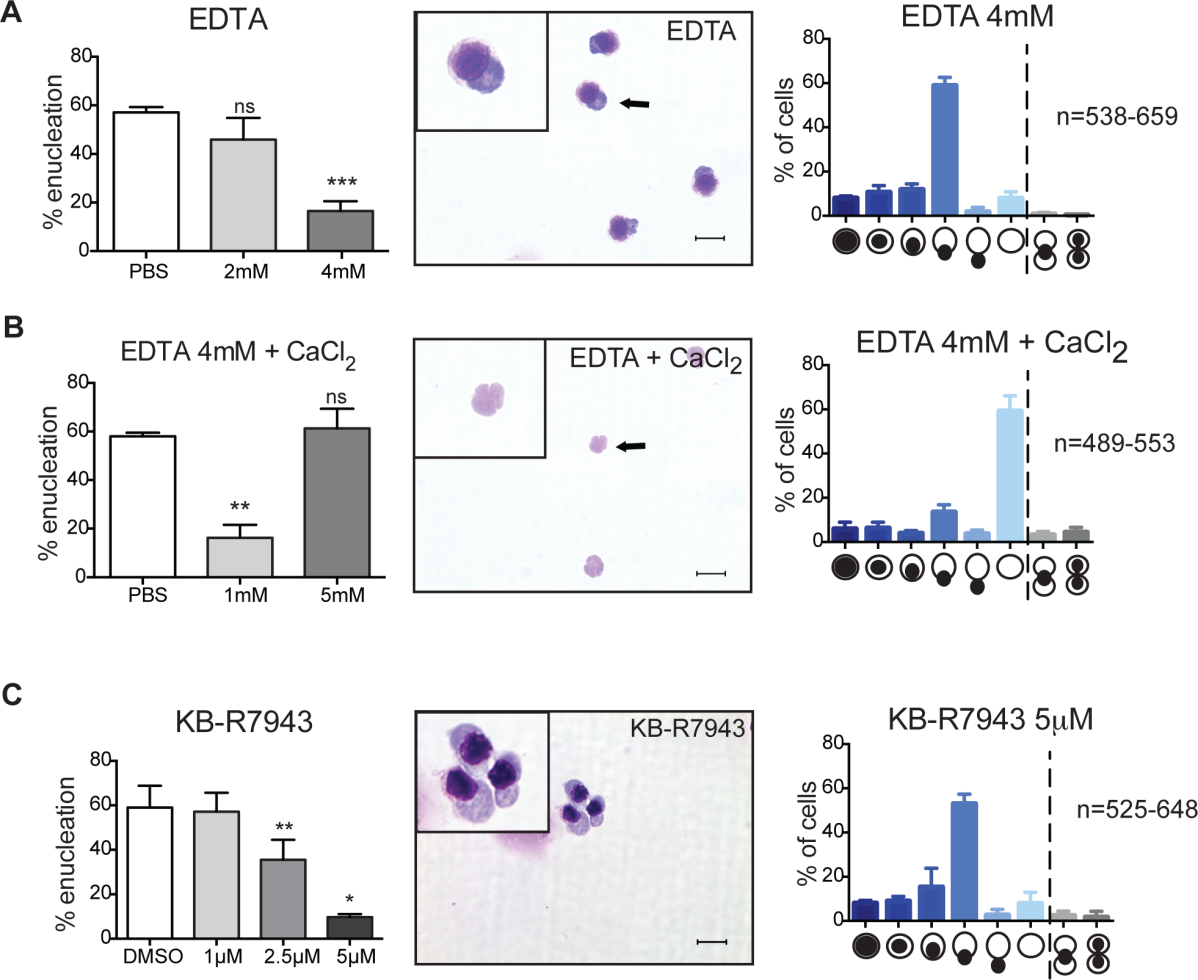
Erythroid enucleation requires uptake of extracellular calcium. **(A-C)** Left panels: Orthochromatic erythroblasts were incubated in the presence of the indicated compounds for 5h. Graphs showing percentages of enucleation in the presence of the indicated compounds at the indicated concentrations. Data are means (+/- SD) of 3–4 independent experiments analyzed using FACS LSR II (*P< 0.05, **P< 0.01, ***P< 0.001, ****P< 0.0001 (paired student’s t-test)). Middle and right panels: Cytospins and quantitative analysis of orthochromatic erythroblasts treated with the indicated compounds. Data are means (+/- SD) of 3 independent experiments. Scale bar = 10μm.

To assess which calcium stores may be mobilized, we examined various known sites of calcium release. During cytokinesis, calcium is released from intracellular stores, such as the endoplasmic reticulum (ER), where activated phospholipase C (PLC) cleaves phosphatidylinositol 4,5 bisphosphate (PIP_2_) into 1,4,5-inositol trisphosphate (IP_3_) (S1 Fig). Calcium is then released from the ER into the cytosol through binding of IP_3_ to the IP_3_-receptor [5, 10]. However, targeting this pathway in orthochromatic erythroblasts did not result in inhibition of enucleation (S1 Fig). Interestingly though, we found that enucleation of isolated orthochromatic erythroblasts was significantly decreased in the presence of the calcium chelator EDTA (Fig 3A). Analysis of cytospun erythroblasts allowed to enucleate in the presence of EDTA revealed that around 60% of the cells were arrested at the stage of nuclear extrusion (Fig 3A). Importantly, adding an excess of CaCl_2_ rescued the block caused by EDTA (Fig 3B). This was also confirmed by analysis of cytospins (Fig 3B), demonstrating that erythroid enucleation requires free extracellular calcium. Similar results were also obtained with EGTA, also a calcium chelator (S2 Fig). These results indicated that, despite the commonality in a requirement for calcium signaling for the two processes of cytokinesis and enucleation they differ in regards to their calcium source.

How do orthochromatic erythroblasts take up extracellular calcium? Differential expression analysis of our RNA sequencing data comparing orthochromatic erythroblast to earlier developmental stages revealed that expression of the potassium-dependent sodium-calcium exchanger (solute carrier family 24, member 1) (NCKX1) was highly up-regulated (~ 4.9 times) (our unpublished data). Western blot data confirmed that NCKX1 protein was expressed in orthochromatic and earlier erythroblast stages, however we did not observe differential expression between theses cell stages as our mRNA analysis initially suggested (S3 Fig). Potassium-dependent sodium-calcium exchangers (NCKXs) are bi-directional transporters of Ca^2+^ that transport sodium (Na^+^) in exchange for Ca^2+^ and potassium (K^+^). For example, Na^+^/Ca^2+^ exchangers in resting platelets function in Ca^2+^ efflux mode (extracellular Na^+^ in exchange for intracellular Ca^2+^), while activation of platelets requires influx of Ca^2+^ via NCKX1 [11]. Since orthochromatic erythroblasts require influx of calcium prior to enucleation, and since NCKX1 is expressed in enucleating cells, we tested the effects of KB-R7934, an inhibitor of reverse Na^+^/Ca^2+^ exchange, on enucleation efficiencies and found that enucleation was significantly inhibited (decreased up to 84%) in the presence of this inhibitor (Fig 3C). Morphological analysis revealed that, similar to other modes of calcium signaling inhibition, enucleation was blocked at the nuclear extrusion step (Fig 3C). Together these results indicate that uptake of extracellular calcium is crucial for nuclear extrusion and that calcium influx may at least partly be regulated by reverse Na^+^/Ca^2+^ exchange. Under normal conditions the major function of Na^+^/Ca^2+^ exchangers is to extrude Ca^2+^ from the cytosol in order to keep cytosolic calcium concentrations low. The reverse mode facilitates calcium intake and is normally the result of alterations in membrane potential or ion gradients [12]. Future experiments are needed to determine what signals or events may induce these changes that result in the uptake of calcium required for nuclear extrusion during the enucleation process.

### Erythroid enucleation requires calmodulin signaling

**Fig 4:**
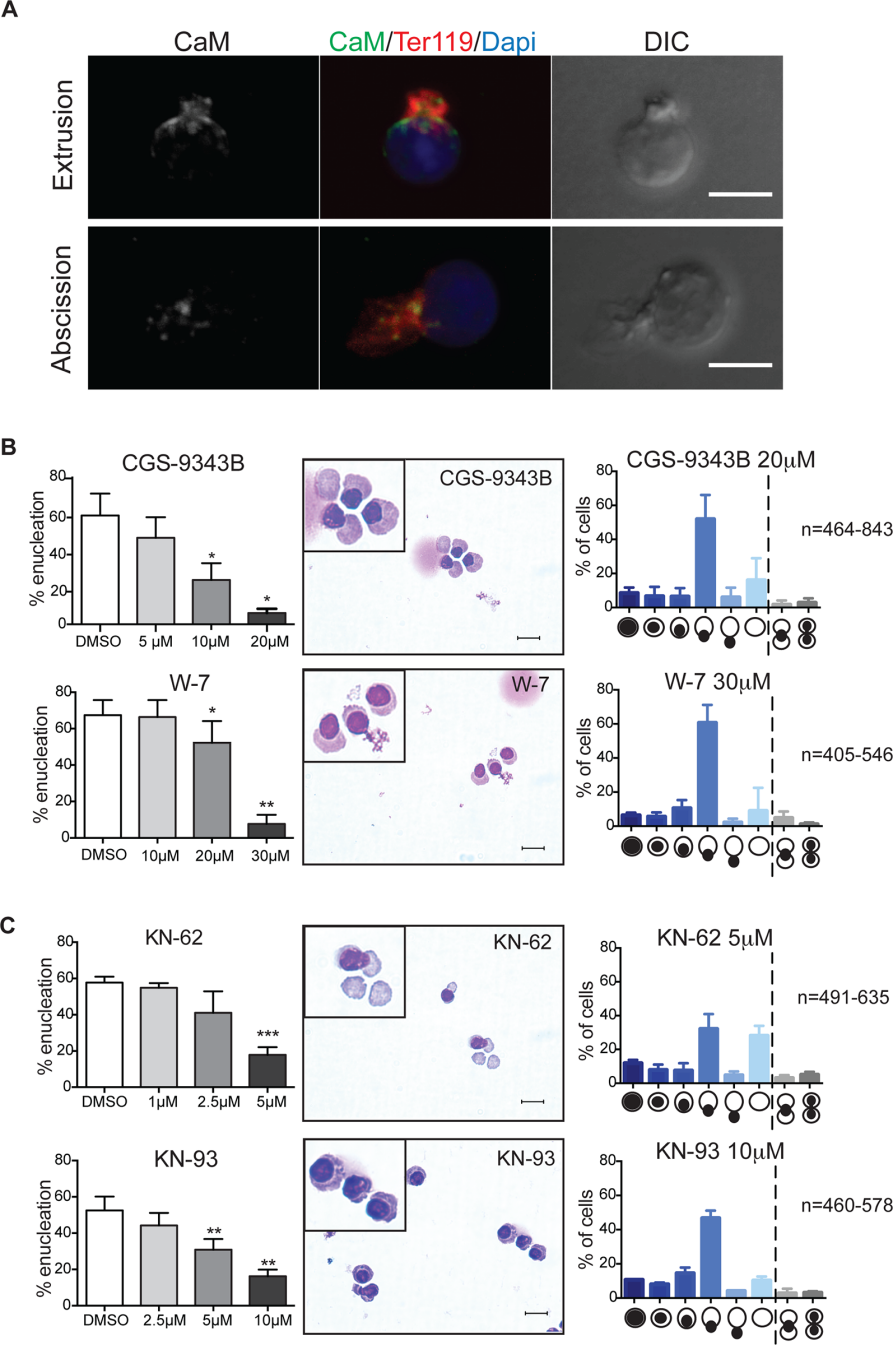
Erythroid enucleation requires calmodulin signaling. **(A)** Immunofluorescence staining for calmodulin (CaM) in orthochromatic erythroblasts at different stages of the enucleation process. Scalebar = 5μm. **(B-C)** Left panels: Orthochromatic erythroblasts were incubated in the presence of the indicated compounds for 5h. Graphs showing percentages of enucleation in the presence of the indicated compounds at the indicated concentrations. Data are means (+/- SD) of 3–4 independent experiments analyzed using FACS LSR II (*P< 0.05, **P< 0.01, ***P< 0.001, ****P< 0.0001 (paired student’s t-test)). Middle and right panels: Cytospins and quantitative analysis of orthochromatic erythroblasts treated with the indicated compounds. Data are means (+/- SD) of 3 independent experiments. Scale bar = 10μm.

**Fig 5:**
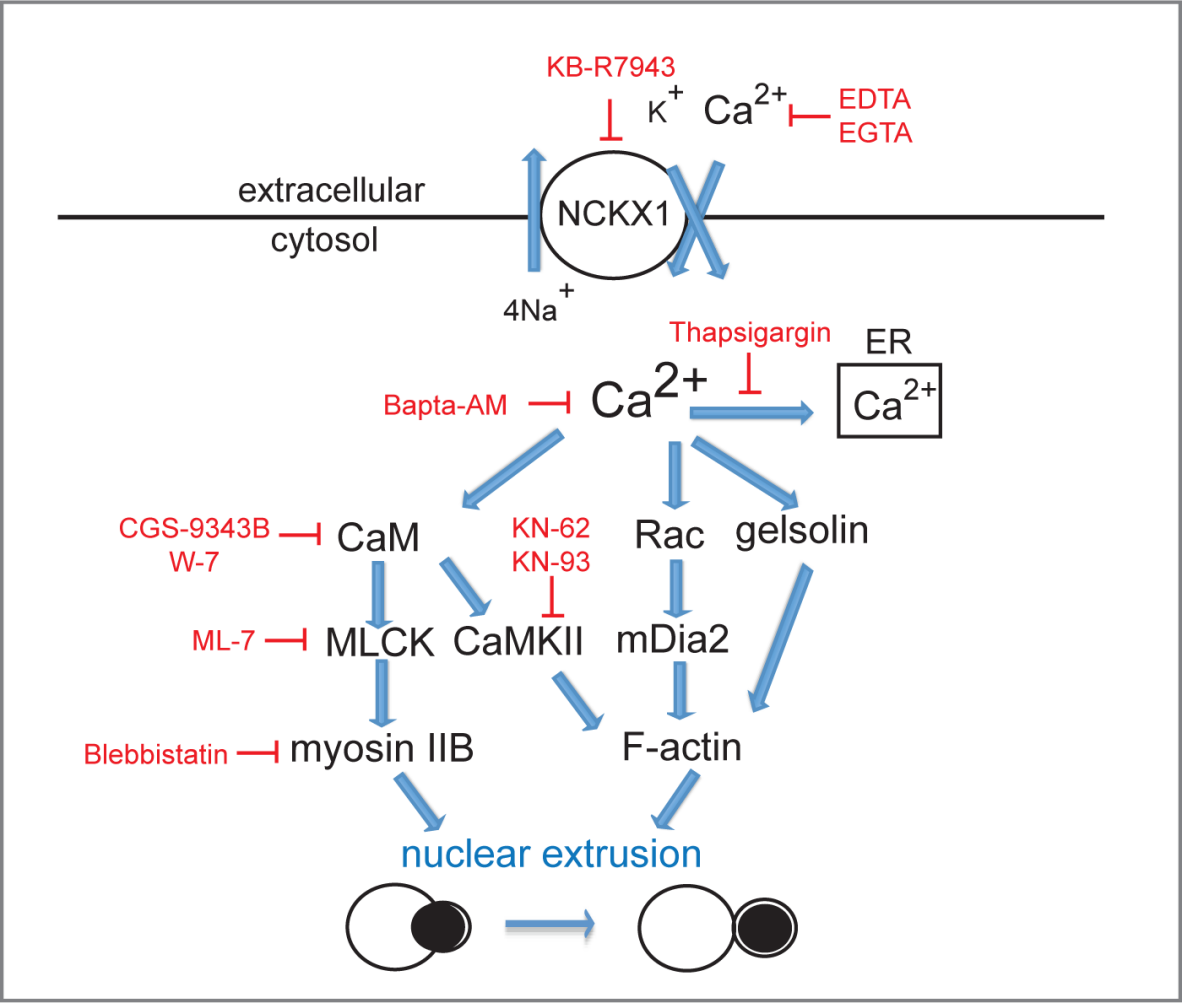
Model of potential roles for calcium in erythroid enucleation. Model of potential actions of calcium during nuclear extrusion. Inhibitors that target calcium-dependent signaling pathways and resulted in arrest of enucleation are shown in red.

Calmodulin (CaM) is a mediator of intracellular calcium signaling. During cytokinesis, CaM localizes to the intercellular bridge [13] and RNAi studies have shown that CaM is crucial for efficient completion of cytokinesis [14]. Given the functional requirement for calcium in the enucleation event, we next explored a role for CaM in erythroid enucleation. Immunofluorescence revealed that CaM localizes to the abscission site between the future red blood cell and the pyrenocyte (Fig 4A). Selective CaM antagonist, CGS-9343B and W-7, the latter also known to inhibit Ca^2+^/CaM-dependent phosphodiesterase [15] and myosin light chain kinase [16], inhibited enucleation in a dose-dependent manner (Fig 4B). Examination of cytospun erythroblasts incubated in the presence of inhibitors targeting CaM activity revealed a morphological block at the nuclear extrusion step of erythroid enucleation, the same step as our previous manipulations of calcium concentrations (Fig 4B). CaM is a regulator of myosin light chain kinase (MLCK), which together with Rho-kinase (ROCK) and citron kinase is important for phosphorylation and activation of myosin II, which also accumulates at the cleavage furrow and is required for proper furrow ingression during cytokinesis [17–20]. Importantly, during enucleation non-muscle myosin IIB localizes to the abscission site between the future pyrenocyte and the future reticulocyte and is thought to be crucial for actomyosin contractility resulting in nuclear extrusion [21]. Here, we confirm these results: in the presence of inhibitors of MLCK (ML-7) and myosin II (Blebbistatin) enucleation is blocked during the nuclear extrusion step (S4 Fig). Our observation that, similar to myosin IIB and F-actin, CaM also localizes to the abscission site during the enucleation event, suggests that myosin II may be a downstream-target of CaM and that this calcium-dependent pathway may be involved in generating contractile forces required for the extrusion of the nucleus (Fig 5). Interestingly, calcium-calmodulin-dependent kinase II (CaMKII), another downstream kinase regulated by CaM can control organization of the actin cytoskeleton through F-actin cross-linking and the rate of polymerization through sequestering of G-actin [22–24]. We therefore also tested two inhibitors (KN-62 and KN-93) of CaMKII, and found that both inhibited enucleation (Fig 4C). Similar to inhibitors targeting its upstream target calmodulin, targeting CaMKII resulted in an arrest of enucleating cells during extrusion of the nucleus (Fig 4C). Together our data indicates that CaM may regulate two different pathways, a CaM/MLCK/myosin II and a CaM/CaMKII pathway, both required for nuclear extrusion upon binding of calcium. Of note, calcium signaling may also coordinately regulate other proteins involved in the enucleation process. For example, gelsolin, a Ca^2+^-activated actin filament has been shown to be critical for terminal erythroid maturation and enucleation [25], suggesting that the transient calcium uptake/calcium flux may be required for extensive gelsolin-dependent actin remodeling required for the enucleation process (Fig 5). Similarly, calcium is thought to regulate translocation and activation of Rac GTPases [26]. Rac has been shown to be important for the formation and contraction of the actomyosin ring during the enucleation process [27]. Intracellular calcium signaling in enucleating cells may thus regulate cytoskeletal changes by directly regulating localization and activation of Rac (Fig 5). If this is the case, it may also be possible that the nuclear segmentation phenotype observed in erythroblasts in the presence of Thapsigargin (Fig 2E) may be the result of hyper-active Rac. In support of this hypothesis, incubating enucleating erythroblasts with both, Thapsigargin and the actin inhibitor Cytochalasin D resulted in a block during nuclear extrusion but completely prevented nuclear segmentation (S5 Fig), suggesting that the segmentation phenotype may be the result of inappropriate positioning of the contractile actin cytoskeleton. These results suggest a potential link between calcium signaling and the actin cytoskeleton, whereby calcium signals may lead to CaMKII- and/or Rac-mediated reorganization of the actin cytoskeleton required for nuclear extrusion. Interestingly, we also observed a segmentation of the nucleus in cytospun erythroblasts allowed to enucleate in the presence of proteasome inhibitors [8], indicating that calcium signaling and the proteasome may coordinately regulate the actin cytoskeleton during the enucleation event. Further studies will be required to test this.

Taken together the data presented in this study show for the first time that erythroid enucleation requires uptake of extracellular calcium and that intracellular calcium signaling through calmodulin is crucial for efficient nuclear extrusion.

## Supporting Information

S1 Fig Calcium release from intracellular stores is not required for erythroid enucleation. **(A)** Schematic of inhibitor targeted pathway. **(B)** Orthochromatic erythroblasts were incubated in the presence of the indicated compounds for 5h. Data are means (+/- SD) of 3 independent experiments analyzed using FACS LSR II (*P< 0.05, **P< 0.01, ***P< 0.001, ****P< 0.0001 (paired student’s t-test)).
(PDF)

S2 Fig Erythroid enucleation requires uptake of extracellular calcium. Orthochromatic erythroblasts were incubated in the presence of the indictaed compounds at the indicated concentrations for 6h. Graphs showing percentages of enucleation in the presence of the indicated compounds at the indicated concentrations. Data are means (+/- SD) of 4 independent experiments analyzed using FACS LSR II (*P< 0.05, **P< 0.01, ***P< 0.001, ****P< 0.0001 (paired student’s t-test)).
(PDF)

S3 Fig NCKX1 expression in terminal differentiating erythroblasts. Polychromatic (P) and orthochromatic (O) erythroblasts were isolated from bone marrow by FACS and subsequently lysed in SDS buffer. Immunoblotting was performed against NCKX1 and β-actin, the loading control.
(PDF)

S4 Fig Erythroid enucleation requires myosin light chain kinase and myosin II activity. Left panels: Orthochromatic erythroblasts were incubated in the presence of the indicated compounds for 5h. Graphs showing percentages of enucleation in the presence of the indicated compounds at the indicated concentrations. Data are means (+/- SD) of 3 independent experiments analyzed using FACS LSR II (*P< 0.05, **P< 0.01, ***P< 0.001, ****P< 0.0001 (paired student’s t-test)). Middle and right panels: Cytospins and quantitative analysis of orthochromatic erythroblasts treated with the indicated compounds. Data are means (+/- SD) of 2 independent experiments. Scale bar = 10μm.
(TIF)

S5 Fig Characterization of nuclear segmentation a result of non-transient intracellular calcium increase. Orthochromatic erythroblasts were incubated in media containing the indicated compounds at the indicated concentration for 5h and subsequently cytospun. For quantitative analysis cells were manually examined (Olympus BX-51 microscope; 100x/1.40 NA oil objective) using the Spot Advanced software (version 4.7)) and assigned a morphological class as per illustration. Data are means (+/- SD) of 3 independent experiments.
(PDF)
