# Supplementary figures and images for: Calcium Signaling Is Required for Erythroid Enucleation
Source: PLoS One. 2016 Jan 5;11(1):e0146201. doi: 10.1371/journal.pone.0146201 (PMC4701494; doi:10.1371/journal.pone.0146201)

S1 Fig

**A**

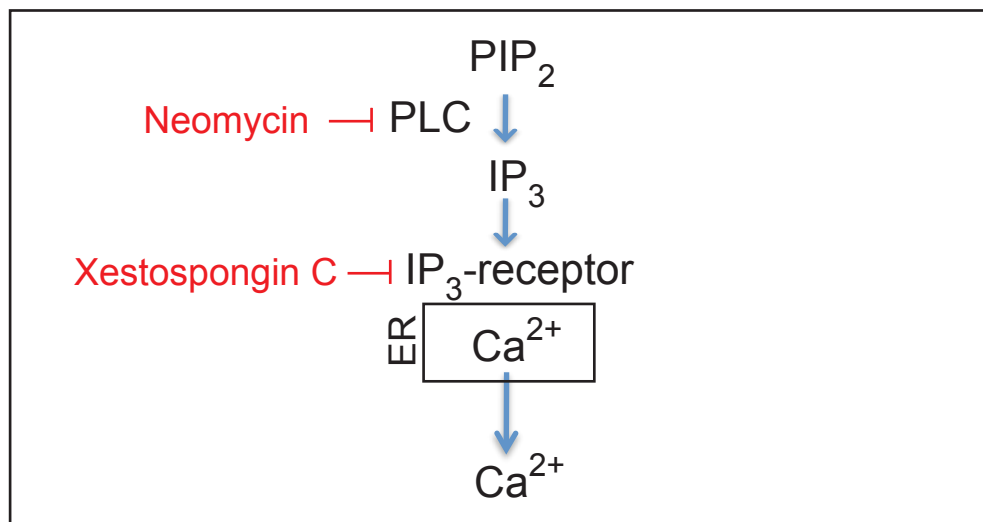

**B**

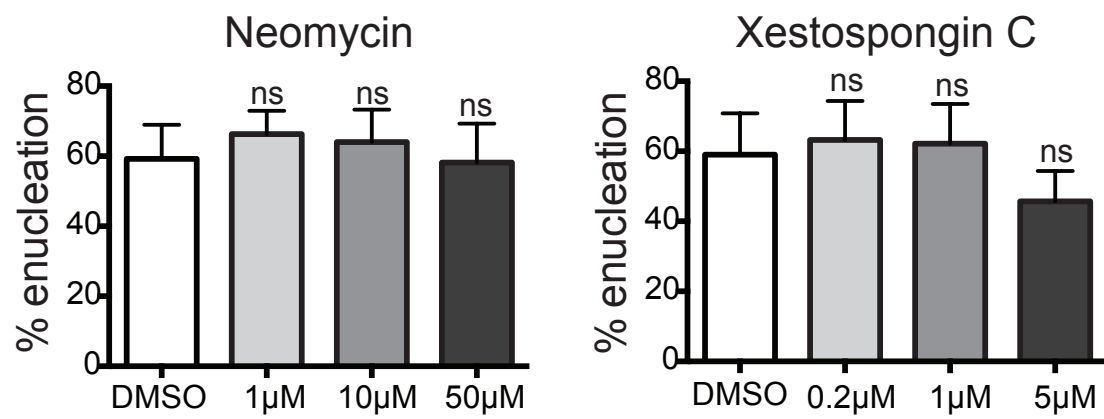

Supplement: S1 Fig — (A) Schematic of inhibitor targeted pathway. (B) Orthochromatic erythroblasts were incubated in the presence of the indicated compounds for 5h. Data are means (+/- SD) of 3 independent experiments analyzed using FACS LSR II (*P< 0.05, **P< 0.01, ***P< 0.001, ****P< 0.0001 (paired student’s t-test)). (PDF) [file pone.0146201.s001.pdf]

S2 Fig

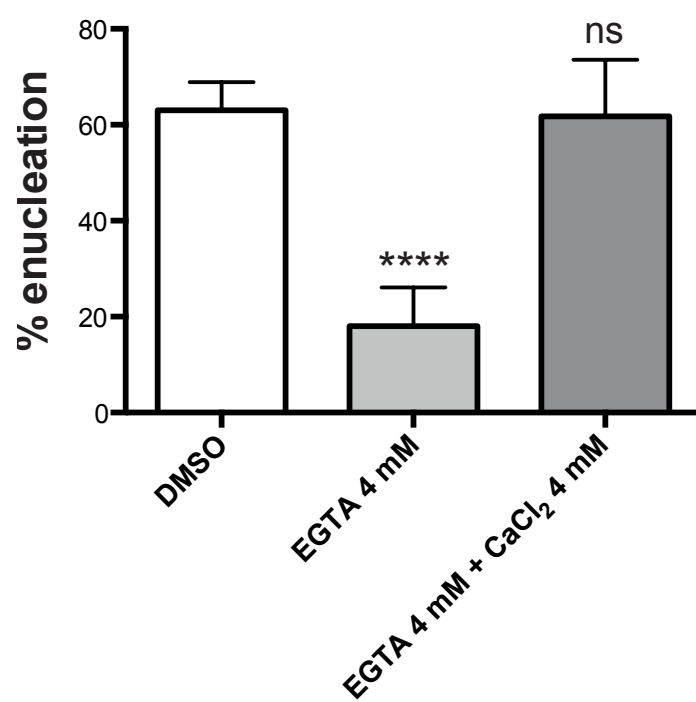

Supplement: S2 Fig — Orthochromatic erythroblasts were incubated in the presence of the indictaed compounds at the indicated concentrations for 6h. Graphs showing percentages of enucleation in the presence of the indicated compounds at the indicated concentrations. Data are means (+/- SD) of 4 independent experiments analyzed using FACS LSR II (*P< 0.05, **P< 0.01, ***P< 0.001, ****P< 0.0001 (paired student’s t-test)). (PDF) [file pone.0146201.s002.pdf]

S3 Fig

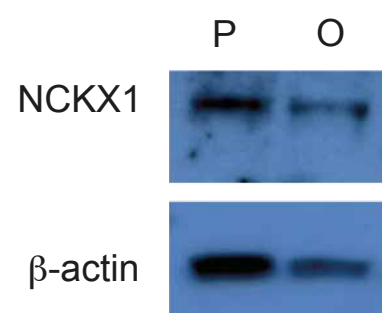

Supplement: S3 Fig — Polychromatic (P) and orthochromatic (O) erythroblasts were isolated from bone marrow by FACS and subsequently lysed in SDS buffer. Immunoblotting was performed against NCKX1 and β-actin, the loading control. (PDF) [file pone.0146201.s003.pdf]

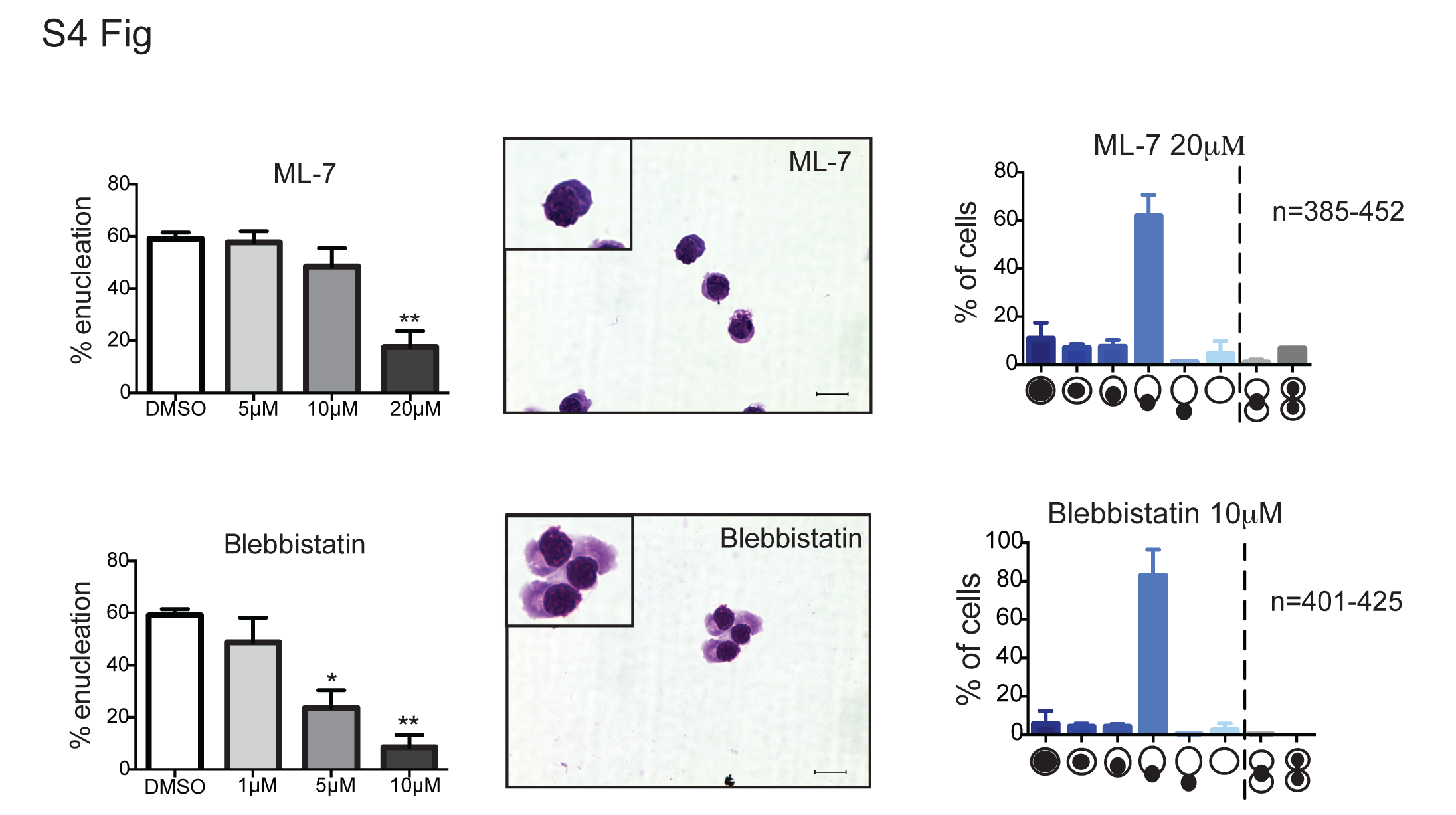

Supplement: S4 Fig — Left panels: Orthochromatic erythroblasts were incubated in the presence of the indicated compounds for 5h. Graphs showing percentages of enucleation in the presence of the indicated compounds at the indicated concentrations. Data are means (+/- SD) of 3 independent experiments analyzed using FACS LSR II (*P< 0.05, **P< 0.01, ***P< 0.001, ****P< 0.0001 (paired student’s t-test)). Middle and right panels: Cytospins and quantitative analysis of orthochromatic erythroblasts treated with the indicated compounds. Data are means (+/- SD) of 2 independent experiments. Scale bar = 10μm. (TIF) [file pone.0146201.s004.tif]

**A**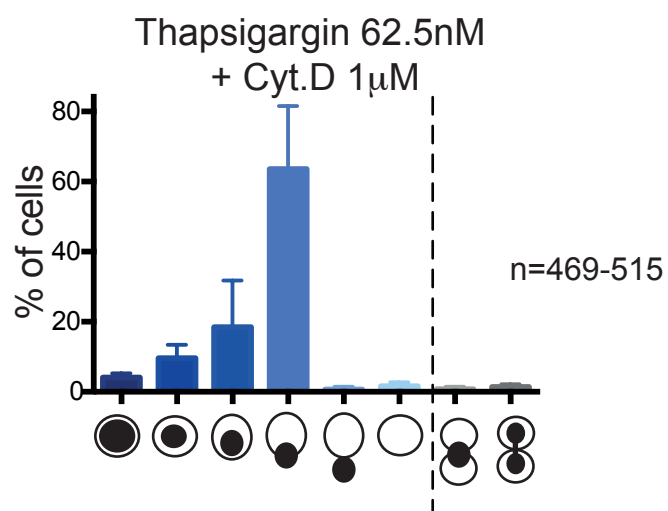

Supplement: S5 Fig — Orthochromatic erythroblasts were incubated in media containing the indicated compounds at the indicated concentration for 5h and subsequently cytospun. For quantitative analysis cells were manually examined (Olympus BX-51 microscope; 100x/1.40 NA oil objective) using the Spot Advanced software (version 4.7)) and assigned a morphological class as per illustration. Data are means (+/- SD) of 3 independent experiments. (PDF) [file pone.0146201.s005.pdf]
